# Novel Interplay between p53 and HO-1 in Embryonic Stem Cells

**DOI:** 10.3390/cells10010035

**Published:** 2020-12-29

**Authors:** Ayelén Toro, Nicolás Anselmino, Claudia Solari, Marcos Francia, Camila Oses, Pablo Sanchis, Juan Bizzotto, Camila Vazquez Echegaray, María Victoria Petrone, Valeria Levi, Elba Vazquez, Alejandra Guberman

**Affiliations:** 1 CONICET, Instituto de Química Biológica de la Facultad de Ciencias Exactas y Naturales (IQUIBICEN), Universidad de Buenos Aires, Buenos Aires C1428EGA, Argentina; ayelentoro@gmail.com (A.T.); nicoanselmino@gmail.com (N.A.); cmsolari@gmail.com (C.S.); marcosgabrielfrancia@gmail.com (M.F.); camilaoses94@gmail.com (C.O.); pabloasanchis@gmail.com (P.S.); juanantoniobizzotto@gmail.com (J.B.); camivazqueze@yahoo.com.ar (C.V.E.); mavipetrone@gmail.com (M.V.P.); vlevi12@gmail.com (V.L.); 2 Departamento de Química Biológica, Facultad de Ciencias Exactas y Naturales, Universidad de Buenos Aires, Buenos Aires C1428EGA, Argentina; 3 Departamento de Fisiología y Biología Molecular y Celular, Facultad de Ciencias Exactas y Naturales, Universidad de Buenos Aires, Buenos Aires C1428EGA, Argentina

**Keywords:** heme oxygenase-1, p53, embryonic stem cells, gene expression regulation

## Abstract

Stem cells genome safeguarding requires strict oxidative stress control. Heme oxygenase-1 (HO-1) and p53 are relevant components of the cellular defense system. p53 controls cellular response to multiple types of harmful stimulus, including oxidative stress. Otherwise, besides having a protective role, HO-1 is also involved in embryo development and in embryonic stem (ES) cells differentiation. Although both proteins have been extensively studied, little is known about their relationship in stem cells. The aim of this work is to explore HO-1-p53 interplay in ES cells. We studied HO-1 expression in p53 knockout (KO) ES cells and we found that they have higher HO-1 protein levels but similar HO-1 mRNA levels than the wild type (WT) ES cell line. Furthermore, cycloheximide treatment increased HO-1 abundance in p53 KO cells suggesting that p53 modulates HO-1 protein stability. Notably, H_2_O_2_ treatment did not induce HO-1 expression in p53 KO ES cells. Finally, SOD2 protein levels are also increased while *Sod2* transcripts are not in KO cells, further suggesting that the p53 null phenotype is associated with a reinforcement of the antioxidant machinery. Our results demonstrate the existence of a connection between p53 and HO-1 in ES cells, highlighting the relationship between these stress defense pathways.

## 1. Introduction

Diverse hostile conditions perturb cell homeostasis, inducing cellular stress that consequently results in gene expression alterations and damage to DNA, RNA, proteins and lipids [1]. Stress stimuli come from both external and internal sources. Extrinsic stress is usually determined by environmental factors that cause tissue and cellular damage. On the other hand, intrinsic types of stress are generated by normal metabolism which produces an accumulation of residual products and reactive metabolites [2]. Such is the case of oxidative stress promoted by multiple stimuli including reactive oxygen species (ROS), products of physiological cell activity originated in many cellular compartments. Although low to moderate levels of ROS are required for various cellular processes such as signal transduction, proliferation, apoptosis and differentiation [3,4,5], the accumulation of ROS has harmful effects on cell homeostasis and on diverse cellular structures and functions. Of note, excessive ROS production is associated with diverse human diseases, inflammation, and aging [6].

Embryonic stem (ES) cells are pluripotent cells that have the ability to differentiate into all the adult cell lineages, and are also characterized by an unlimited replication potential [7,8]. Stress mitigation is crucial in pluripotent stem cells due to their essential role in development and the risk associated with malignant transformation. In addition, multiple pathways related to stress response and repair are upregulated in these cells, therefore ensuring their genomic stability [2,9,10].

Particularly, pluripotent stem cells harbor lower ROS levels due to their exacerbated antioxidant mechanisms characterized by gene induction and activation of multiple detoxifying enzymes [11]. However, fine-tuned ROS levels are involved in signaling pathways promoting, for example, specific differentiation processes. Therefore, intracellular redox status regulates the balance between self-renewal and differentiation [12,13,14,15,16]. Consequently, oxidative stress control is highly relevant for self-renewal and pluripotency maintenance of pluripotent stem cells [17].

The transcription factor (TF) and tumor suppressor p53 plays a vital role in the cell by controlling cellular response to multiple types of harmful stimulus, including oxidative stress. Under transient and mild oxidative stress conditions, p53 holds an antioxidant function and contributes to maintain low ROS levels; however, under severe stress, p53 has pro-oxidant actions [18]. The main mechanisms by which this protein exerts its actions include inhibition of cell proliferation, induction of cell differentiation, modulation of metabolism, and the onset of apoptosis or cellular senescence [19]. Regarding ROS modulation by p53 in the context of stem cells and development, it was reported that p53 fine-tunes endogenous ROS levels to ensure the proper prenatal neurogenesis [20]. Additionally, p53 maintains the pool of hematopoietic stem cells by reducing ROS levels [21]. In pluripotent stem cells, p53 activates the expression of antioxidant genes, thus reducing the levels of oxidative stress and contributing to genomic stability [22]. Particularly, the primary role of p53 in ES cells is to promote differentiation in response to DNA damage or developmental signals [23]. For instance, p53 represses the expression of Nanog, a key pluripotency TF, thus inducing ES cells to differentiate into cell types that can efficiently execute cell death programs [24]. On the other hand, it has also been demonstrated that p53 is required for ES cells self-renewal [25]. This apparently contradictory evidence highlights the complexity of p53 function in ES cells.

A highly relevant component of the antioxidant system is heme oxygenase-1 (HO-1), encoded by *Hmox1* gene, an integral protein of the endoplasmic reticulum (ER) that exhibits cytoprotective and anti-inflammatory functions [26]. In addition to HO-1 cytoprotective ability, this protein is also involved in embryo development and stem cells differentiation [27]. Interestingly, *Hmox1* contains p53 response elements [28] and this TF promotes cell survival by *Hmox1* induction under oxidative stress conditions [29]. The main TF that promotes *Hmox1* expression is the nuclear factor erythroid 2-related factor 2 (NRF2), that coordinates the basal and stress-induced activation of several cytoprotective genes, controlling key components of the cellular antioxidant system [30]. Both NRF2 and p53 regulate the expression of proteins involved in the protection against oxidative stress, and it has been suggested that there is a crosstalk between them [31,32], even though this interplay is not completely understood. Moreover, a link between p53 and NRF2 was described in multiple tumorigenic scenarios like lung and breast cancer [33,34]. Furthermore, NRF2 deficits are buffered by compensatory increases in p53 signaling, and striking ROS-dependent phenotypes arise when both pathways are perturbed in breast-mammary epithelia [32]. In the context of stem cells, NRF2 overexpression protects mesenchymal stem cells against cell death caused by oxidative stress and preserves multi-differentiation potential [35]. Moreover, NRF2 plays a crucial role in the maintenance of mesenchymal stem cells properties through p53–SIRT1 axis regulation [36]. Remarkably, NRF2 controls the self-renewal and pluripotency of human ES cells and is required for pluripotency reestablishment during induced pluripotent stem (iPS) cells generation [13]. Furthermore, it was found that HO-1 modulation affects both, the cellular reprograming efficiency and the capability of pluripotent stem cells to spontaneously differentiate to cardiac lineages [37]. Interestingly, it was suggested that HO-1 function during fibroblasts reprograming could be related to p53 downregulation [37]. This evidence highlights the existence of an interesting and complex connection between p53 and HO-1 in pluripotent stem cells that has not been fully described yet.

In this work, we found that p53 knockout (KO) ES cells have higher HO-1 protein levels compared to the wild type (WT) cell line. Our results suggest the existence of a p53-dependent negative modulation of HO-1 protein stability and that p53 null phenotype is related with an altered ROS homeostasis in ES cells, associated with a reinforcement of the antioxidant machinery at the post-translational level. Furthermore, we found that HO-1 increases during differentiation in both WT and KO ES cells, suggesting a possible connection between HO-1 and the exit of the pluripotent state or differentiation progression. As a whole, our results evidence a regulatory crosstalk between these two stress pathways in ES cells.

## 2. Materials and Methods

### 2.1. Cell Culture and Treatments

The W4 ES cell line was provided by the Rockefeller University Core Facility and W4 p53 KO ES cell line was previously generated by CRISPR/Cas9 technology in our lab [38]. Cells were routinely cultured in 2i/LIF ES medium consisting of DMEM, Glutamax (2 mM), MEM NEAA (100 mM), 2-mercaptoethanol (0.1 mM), penicillin (100 U/mL), streptomycin (100 mg/mL) and FBS (15%, Gibco, Paisley, UK), LIF, PD0325901 (1 μM, Tocris, Bristol, UK) and CHIR 99,021 (3 μM, Tocris). Cells were plated on bovine gelatin (0.1%, Sigma, St. Louis, MO, USA) coated dishes at 37 °C in a 5% CO_2_ (*v*/*v*) incubator and passaged every three days. To induce HO-1 expression, cells were pre-treated or treated with hemin (20 μM, Sigma) during 20 h. Cell viability and proliferation were evaluated using MTT (Sigma) and Crystal Violet. To inhibit protein synthesis cells were treated with cycloheximide (10 μM, Sigma) during 2 or 7 h. To induce differentiation cells were cultured in the absence of LIF and 2i for 48 h. To evaluate the effect of H_2_O_2_ on HO-1 induction cells were exposed to H_2_O_2_ (100 μM) during 4 h.

### 2.2. Gene Expression Analysis

Gene expression was analyzed by reverse transcription real-time quantitative PCR (RT-qPCR), immunofluorescence (IF) and/or Western blot (WB) as previously described [39], using the following primers and antibodies: primers: *Gapdh* (Forward TGCCAAGGCTGTGGGCAAGG, Reverse CGAAGGTGGAAGAGTGGG), *Hmox1* (Forward AAGAGGCTAAGACCGCCTTC, Reverse GCATAAATTCCCACTGCCAC) and *Sod2* (Forward AAGCACCACGCGGCCTACG, Reverse CCATTGAACTTCAGTGCAGGCTG); antibodies: anti-HO-1 (Abcam, Cambridge, UK, ab 13248), anti-NRF2 (Santa Cruz, Minneapolis, MN, USA, SC-722), anti-pS40 NRF2 (Abcam, ab76026), anti-SOD2 (Santa Cruz, sc-137254), anti-GAPDH (Santa Cruz, SC-32233), anti-mouse AlexaFluor 555 (Invitrogen, Waltham, MA, USA, A31570), anti-mouse IgG-HRP (Invitrogen, G-21040).

### 2.3. Complementation Assay

W4 p53 KO ES cells were seeded on gelatin-coated coverslips in 24-well plates and transfected with 300 ng of p53-GFP expression vector (kindly provided by Dr. Martin Monte, IQUIBICEN/Universidad de Buenos Aires). Transfection was carried out as previously described [40]. 48 h after transfection, HO-1 was visualized by immunofluorescence and nuclei were counterstained with DAPI. Transfected cells were identified by GFP fluorescence detection.

### 2.4. Nuclear-Cytoplasmic Ratio Estimation

HO-1 immunofluorescence (IF) images were acquired in a wide-field Olympus IX71 microscope equipped with an EXi Aqua Bio-Imaging Microscopy Camera (Qimaging, Surrey, BC, Canada). The mean intensity of HO-1 staining in nuclei and cytoplasm was measured in wide-field images since it was easier to delimit the cytoplasmic region of single cells in comparison to confocal images. Nuclei were identified by DAPI staining and a cytoplasm mask was constructed by thresholding the images obtained in the HO-1 channel and subtracting the region corresponding to nuclei. Nuclei vs. cytoplasm mean intensity was fitted with a linear equation weighted by the number of cells on each field.

### 2.5. Bioinformatics Analysis

The Stemformatics web tool [41] was used to evaluate *HMOX1* and *TP53* gene expressions in different human pluripotent stem cell lines subjected to both non-directed and directed differentiation protocols and in human somatic cells subjected to reprograming (Table 1).

### 2.6. Flow Cytometry

ES cells were plated in a 6-well plate (120,000 cells/well) using ES cell medium. 24 h after cells were trypsinized, resuspended with PBS and incubated with 6 µM 5,6-clorometil-2′7′-dichloro dihydro-fluorescein diacetate, acetyl ester (CM-H2DCFDA; Invitrogen) for 1 h at 37C. H2DCFDA levels were measured by flow cytometry and analyzed with the FlowJo 7.6 software. Experiments with NpFR1 and NpFR2 probes were performed as previously described [51,52].

### 2.7. Statistical Analysis

Experimental results are presented as mean ± standard error of the mean (SEM). Statistical comparisons were performed using Student´s *t*-Test or randomized block design ANOVA followed by a Tukey post-test for at least three biological replicates using Infostat or GraphPad Prism softwares. In all cases, residuals fitted normal distribution, assessed by Shapiro-Wilks test, and homogeneity of variance, using Levene test. *p* values < 0.05 were considered significant.

## 3. Results

### 3.1. HO-1 Protein Levels Are Boosted in p53 Knockout ES Cells

**Figure 1:**
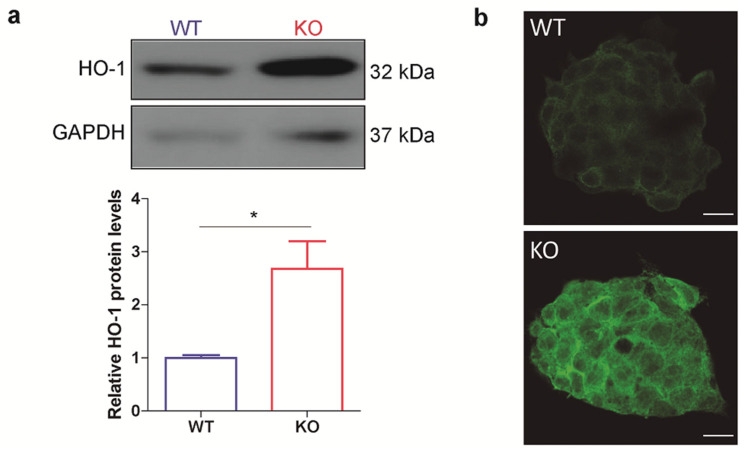
HO-1 protein levels are boosted in p53 knockout ES cells. WT and p53 KO ES cells were cultured under standard conditions. (**a**) HO-1 protein levels were analyzed by Western blot, upper panels show representative blots. GAPDH was used as the loading control. Lower panels show HO-1/GAPDH band densitometry relative to WT ES cells. Bars represent the mean ± SEM of three independent experiments. Asterisks indicate significant differences between cell lines (*, *p* < 0.05). (**b**) HO-1 levels were analyzed by Immunofluorescence. Representative confocal images of ES cell colonies are shown. The green pseudocolor represents HO-1 specific immunostaining, scale bars: 10 µm.

Based on the role of p53 in the cellular defense system and the relationship between p53 and NRF2 mentioned above, we decided to focus our study on the protein HO-1, one of the most relevant targets of NRF2, which is involved in stem cells differentiation and reprograming. To explore p53 and HO-1 interplay in ES cells, we first compared HO-1 protein levels between a p53 null ES cell line and the corresponding WT parental ES cell line. The p53 KO ES cell line was previously generated in our lab by CRISPR/Cas9 technology. It is homozygous for an insertion of a single nucleotide, which induced a frameshift starting on amino acid 17 and a non-sense mutation on exon 4. This cell line displays normal morphology, cell-cycle distribution and similar expression of pluripotency markers compared to the parental cell line [38]. Notably, the analysis of HO-1 protein levels by WB and IF showed higher HO-1 expression in p53 KO ES cells compared to WT (Figure 1a,b). To restore p53 expression in p53 KO ES cells, we performed a complementation assay and we observed that p53-GFP transfected cells displayed lower HO-1 protein levels than non-transfected cells within the same field (Supplementary Figure S1), strongly suggesting that p53 modulates HO-1 levels and further supporting our results.

**Figure 2:**
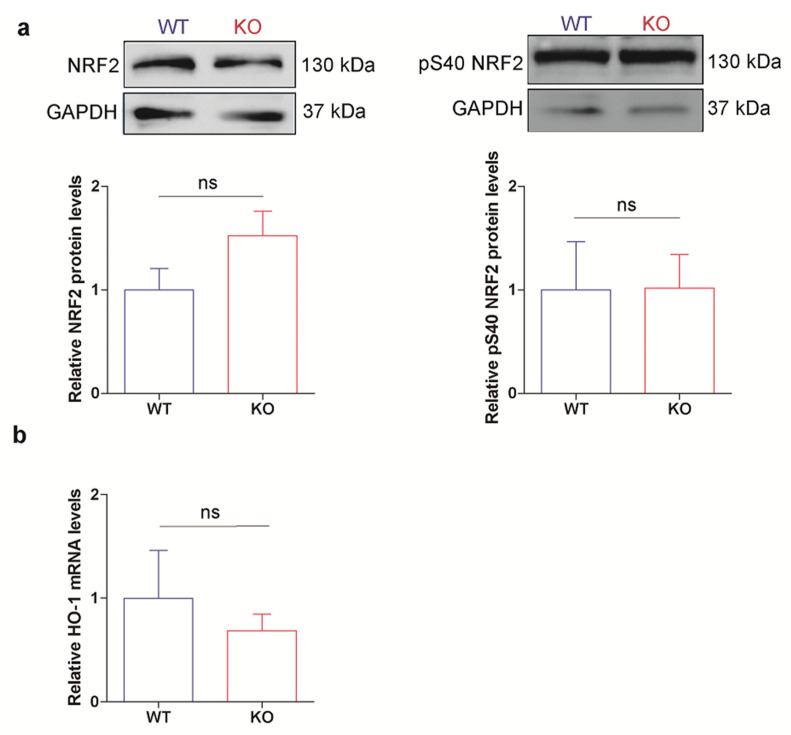
Increased HO-1 expression is not regulated at the transcriptional level. WT and p53 KO ES cells were cultured under standard conditions. (**a**) NRF2 and pS40 NRF2 levels were analyzed by Western blot. Upper panels show representative blots. GAPDH was used as loading control. Lower panels show NRF2 and pS40 NRF2/GAPDH band densitometry relative to WT ES cells. Bars represent the mean ± SEM of three independent experiments. (**b**) HO-1 mRNA levels were analyzed by RT-qPCR, normalized to the housekeeping gene *Gapdh* and referred to WT ES cells. Bars represent the mean ± SEM of three independent experiments. Non-significant (ns) differences between cell lines were observed.

To explore whether NRF2 mediates the exacerbation of HO-1 expression in p53 KO ES cells, we analyzed its status in our experimental system. We found no significant differences between p53 KO and WT ES cells in both total NRF2 and NRF2 phosphorylated at Ser 40 (pS40 NRF2), a post-translational modification (PTM) associated with the activation of this TF [53] (Figure 2a). These results suggest that the differences found in HO-1 levels between these cell lines are not regulated at the transcriptional level. In agreement, no significant changes in HO-1 mRNA levels were observed in p53 KO and WT ES cell lines (Figure 2b), supporting that these boosted HO-1 levels are not due to differential transcriptional regulation and suggesting that a post-transcriptional mechanism might be involved. We hypothesize that the higher HO-1 protein levels found in p53 KO ES cells could probably be due to a cellular strategy to mitigate the stress generated by the lack of p53, in order to restore homeostasis and maintain cellular wellness.

### 3.2. HO-1 Half-Life Is Longer in p53 KO ES Cells

**Figure 3:**
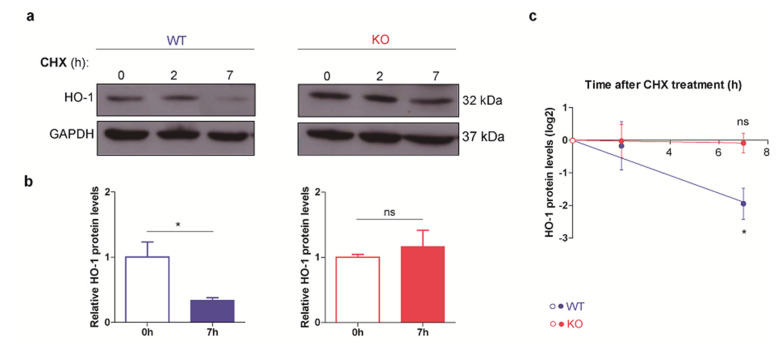
HO-1 half- life is longer in p53 KO embryonic stem (ES) cells. WT and p53 KO ES cells were treated with 10 µM cycloheximide (CHX) during 2 and 7 h. HO-1 levels were analyzed by Western blot. (**a**) Representative blots of HO-1 in WT and p53 KO ES cells. GAPDH was used as loading control. (**b**) HO-1/GAPDH band densitometry of vehicle control (DMSO: 0 h) and CHX treatment (7 h). Bars represent the mean ± SEM of three independent experiments. (**c**) Time-dependent HO-1 expression plot. Dots represent the mean ± SEM of three experiments. Asterisks indicate significant differences compared to the control (*, *p* < 0.05).

To explore whether higher HO-1 levels in p53 KO ES cells were a consequence of a differential post-transcriptional regulation in this cell line, we studied HO-1 half-life. We treated WT and KO ES cells with the commonly used protein synthesis inhibitor cycloheximide (CHX) during 2 or 7 h and evaluated HO-1 protein levels by WB. Interestingly, we found that HO-1 protein levels were less sensitive to CHX treatment in p53 KO ES cells than in WT (Figure 3a), suggesting that the lack of p53 promotes a higher HO-1 stability. We plotted the relative amounts of HO-1 levels for each ES cell line at 7 h of CHX treatment, as at this time point we could detect the differences between KO and WT ES cells. As shown in Figure 3b,c, HO-1 protein levels greatly decreased after CHX treatment in WT ES cells, while in p53 KO ES cells HO-1 protein levels remained similar to the control condition. Subsequently, to rule out the possibility that the observed effect was due to the higher HO-1 basal levels in p53 KO ES cells, we evaluated the effect of CHX treatment in WT cells pre-treated with hemin, a well-known specific HO-1 inducer. The experimental setup of hemin treatment conditions is shown in Supplementary Figure S2a–c. No differences were detected in HO-1 decay rate when comparing basal HO-1 and hemin-induced HO-1 in WT ES cells (Supplementary Figure S2d,e). These results indicate that the selected time-points allowed us to detect HO-1 decay even for high protein levels similar to those observed in p53 KO cells, strongly supporting that the effect in p53 KO ES cells described above was a consequence of a greater HO-1 stability and not due to their higher basal HO-1 levels. Interestingly, p53 KO ES cells showed similar HO-1 stability when cultured with or without hemin and such stability was higher in KO than in WT ES cells since 7 h of CHX treatment are not enough to detect a decrease in HO-1 levels in KO cells (Supplementary Figure S2e). These results suggest that an unknown mechanism dependent on p53 promotes HO-1 protein degradation in ES cells.

### 3.3. p53 KO ES Cells Exhibit Higher HO-1 Cytoplasmic Levels

**Figure 4:**
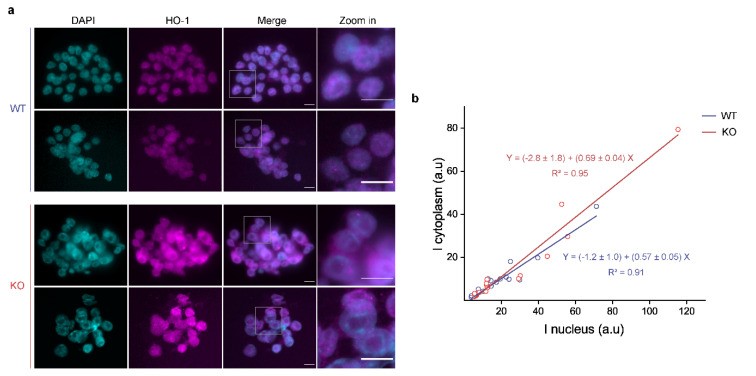
p53 KO ES cells exhibit higher HO-1 cytoplasmic levels. Wild type (WT) and p53 KO ES cells were cultured under standard conditions and HO-1 protein levels were analyzed by immunofluorescence. (**a**) Representative fluorescence microscopy images of WT and KO ES cells. Scale bars: 10 µm. (**b**) HO-1 immunofluorescence intensity of nuclei vs. cytoplasm for WT (blue) or KO (red) ES cells. The equations shown in the figure were obtained by a linear fit of the data, with a statistical weight corresponding to the number of cells in each field.

The stability of multiple proteins is often associated with PTMs and/or interactions with other proteins that promote or interfere the protein degradation [54,55]. Moreover, protein subcellular organization is highly modulated and strongly influences regulatory interactions. Although canonical HO-1 localization is cytoplasmic, this protein was also reported to localize in the nucleus [56,57]. Thus, we explored HO-1 subcellular distribution in our experimental system. Cytoplasmic and nuclear HO-1 localization was assessed by epifluorescence microscopy of HO-1 immunostaining in WT and KO ES cells (Figure 4a). The quantification of HO-1 distribution revealed that the nuclear-cytoplasmic ratio (N/C ratio) was lower in KO cells (Figure 4b), indicating a relatively lower proportion of HO-1 in the nucleus in comparison to the cytoplasm. We detected the same pattern of HO-1 localization by confocal microscopy (Supplementary Figure S3). Further research would be required to explore if HO-1 subcellular localization modulates its stability and whether it is associated with an altered redox status in ES cells.

### 3.4. HO-1 Increases during Differentiation in WT and p53 KO ES Cells

**Figure 5:**
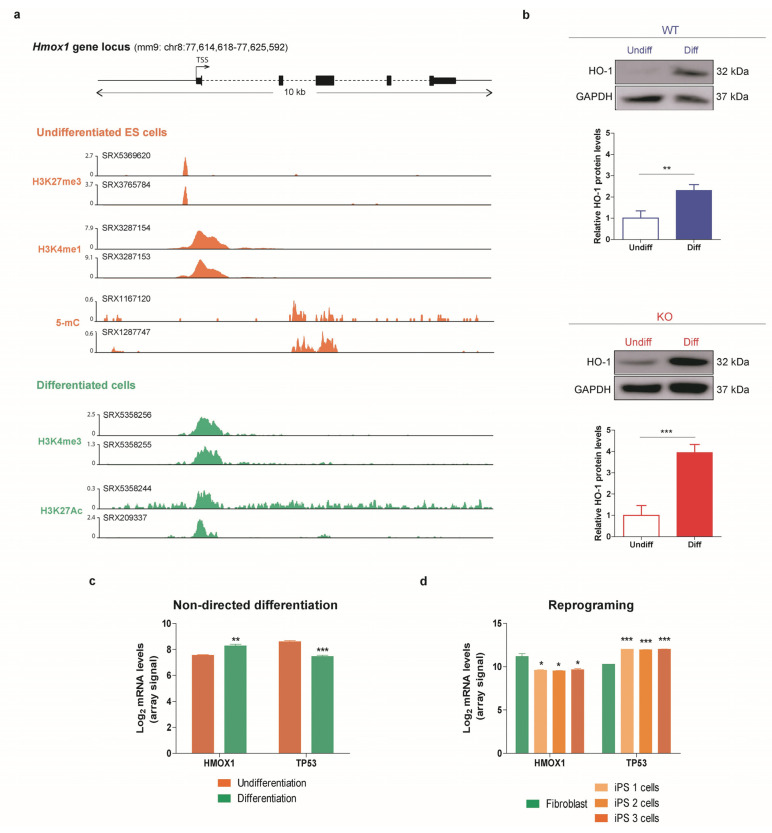
HO-1 increases during differentiation in WT and p53 KO ES cells. (**a**) Analysis of the indicated histone modifications and Cytosine methylation (5-mC) present in *Hmox1* locus in undifferentiated ES cells and ES cells subjected to differentiation protocols (differentiated cells) using the public ChIP-seq data available in Chip Atlas data-mining platform. Dataset identification numbers of each experiment are shown at the top of the y-axis. (**b**) WT and KO ES cells were induced to differentiate by 2i/LIF withdrawal for 2 days (Diff) and HO-1 protein levels were evaluated by Western blot. Upper panels show representative blots. GAPDH was used as loading control. Lower panels show HO-1/GAPDH band densitometry relative to the control condition (Undifferentiated). Bars represent the mean ± SEM of three independent experiments. Asterisks indicate significant differences when compared to the control condition (Undifferentiated) (**, *p* < 0.01; ***, *p* < 0.001). (**c**) Microarray data analysis of *HMOX1* and *TP53* gene expression in undifferentiated human ES and in human ES cells subjected to a non-directed differentiation protocol. (**d**) Microarray data analysis of *HMOX1* and *TP53* gene expression during human fibroblast reprograming into iPS cells. Bars represent the mean ± SEM of each experiment. Asterisks indicate significant differences between undifferentiated (orange) and differentiated (green) cells (*, *p* < 0.05; **, *p* < 0.01; ***, *p* < 0.001).

Based on the evidence that HO-1 is involved in stem cells differentiation [37,58,59,60], we analyzed the HO-1 expression in ES cells during this process. First, we exploited ChIP Atlas data-mining platform [61] to investigate histone modifications and the binding of multiple transcriptional regulators to *Hmox1* locus in undifferentiated ES cells and in ES cells subjected to differentiation protocols. The examination of the integrated data showed that several proteins associated with transcription repression bind to the *Hmox1* regulatory region in undifferentiated ES cells (Supplementary Figure S4a), such as EZH2 and SUZ12, both components of the Polycomb repressive complex, and MBD3 which binds to methylated CpG on DNA, among others. Interestingly, these negative regulators are not associated with *Hmox1* in ES cells that were induced to differentiate. Furthermore, the analysis of histone modifications in undifferentiated ES cells displayed two marks commonly associated with gene repression, trimethylation of H3K27 and methylation of H3K4. In these cells, we also detected the repressive cytosine modification, 5-methylcytosine (5-mC). Further, trimethylation of H3K4 and acetylation of H3K27, two marks related to gene induction, were found in differentiated ES cells (Figure 5a). These results support our findings which demonstrate that HO-1 gene expression increases during ES cells differentiation (Figure 5b). Based on this evidence, our next purpose was to explore HO-1 protein levels in p53 KO ES cells during differentiation. For this aim, we studied whether HO-1 expression was sensitive to the differentiation signals in a p53 null context. As shown in Figure 5b, HO-1 expression was also induced in p53 KO cells along differentiation.

Additionally, we performed a bioinformatics analysis to evaluate HO-1 expression during human ES cells differentiation exploiting publicly available data from high throughput microarrays and RNA-sequencing (RNA-seq) experiments. We found that HO-1 mRNA levels increase during embryo development (Supplementary Figure S4b) and in both non-directed (Figure 5c) and directed (Supplementary Figure S4c) in vitro differentiation protocols. Interestingly, p53 shows the opposite behavior as its mRNA levels decrease during differentiation. In parallel, we examined HO-1 expression during reprograming of human somatic cells into iPS cells. Our analysis showed a significant downregulation of HO-1 mRNA levels through this cellular process (Figure 5d). As a whole, these results highlight the relevance of HO-1 role during ES cells differentiation and its negative association with p53.

### 3.5. HO-1 Is Not Induced in Oxidant Conditions in p53 KO ES Cells Consistent with an Adapted Stress Response

**Figure 6:**
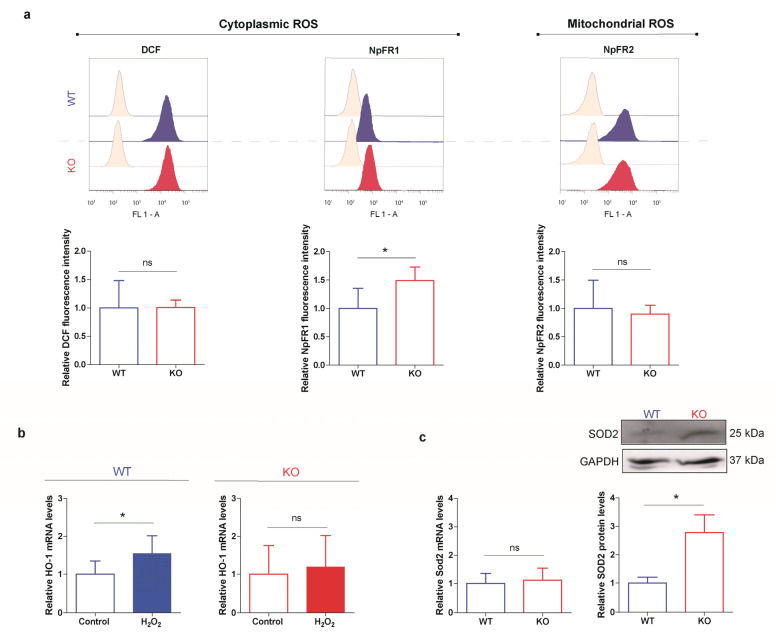
HO-1 is not induced in oxidant conditions in p53 KO ES cells consistent with an adapted stress response. WT and p53 KO ES cells were cultured under standard conditions. (**a**) ROS levels were measured by flow cytometry with H2DCF-DA, NpFR2 and NpFR1 probes. Upper panels show representative histograms and lower panels show the relative fluorescence intensity for each probe. Bars represent the mean ± SEM of three independent experiments. (**b**) WT and KO ES cells were treated with 100 µM H_2_O_2_ during 4 h and HO-1 mRNA levels were analyzed by RT-qPCR. Bars represent the mean ± SEM of four independent experiments. (**c**) *Sod2* mRNA and protein levels were analyzed by RT-qPCR and Western blot, respectively. In RT-qPCR HO-1 or *Sod2* levels were normalized to the housekeeping gene *Gapdh* and referred to the corresponding control (untreated control for (**b**), WT ES cell line for (**c**)). In Western blots GAPDH was used as loading control. Bars represent the mean ± SEM of three independent experiments. Asterisks indicate significant differences when comparing to the corresponding control condition (*, *p* < 0.05).

We reasoned that the lack of p53 and the enhanced HO-1 expression could impact on the redox balance of the p53 KO ES cell line. To evaluate this scenario, we first investigated ROS status by measuring their levels with the commonly used H2DCF-DA probe. As shown in the left panel of Figure 6a, intracellular ROS levels were similar in both cell lines. Furthermore, we studied ROS levels with two fluorescent redox sensors that feature higher sensitivity: NpFR1, a flavin molecule that is almost non-fluorescent in its reduced form and its oxidation gives rise to a 125-fold increase in fluorescence, and NpFR2, a mitochondrial-targeted fluorescent redox sensor which can reversibly measure changes in the mitochondrial redox environment [51,52]. While we did not observe any changes using NpFR2, NpFR1 probe allowed detection of slightly higher cytoplasmic ROS levels in p53 KO cells (Figure 6a, middle and right panels) suggesting a mild difference between cell lines. Then, we evaluated the effect of H_2_O_2_ treatment, commonly used as an oxidant condition, on HO-1 gene induction and we found that while in WT ES cells HO-1 mRNA levels increased as expected, they were not modified in KO ES cells (Figure 6b). These results further evidence an altered response of the cells undergoing oxidative stress conditions. However, they might also indicate that the cytotoxic effects of H_2_O_2_ treatment could be lower in the p53 KO cell line, probably due to the endogenous higher HO-1 levels observed in these cells. Finally, to extend our findings, we explored the expression of Superoxide dismutase 2 (SOD2), a relevant antioxidant enzyme transcriptionally regulated by NRF2 and pluripotency TFs in ES cells [62]. Notably, no differences in *Sod2* transcripts were detected in both ES cell lines; however, its protein levels were boosted in p53 KO ES cells (Figure 6c), similar to the results found for HO-1. These data suggest that p53 null phenotype is related to an altered ROS homeostasis in ES cells, which is also associated with a reinforcement of the antioxidant machinery regulated at the post-translational level.

## 4. Discussion

The present work demonstrates for the first time, a regulatory crosstalk between HO-1 and p53 in ES cells. Another remarkable conclusion that emerges from these data is that HO-1 expression increases during differentiation suggesting an important role of this protein in stem cells biology. Additionally, the lack of p53 correlates with increased HO-1 protein levels presumably as a cellular mechanism to maintain redox homeostasis. This study also highlights that p53 modulates HO-1 expression at the post-transcriptional level, emphasizing the connection between these proteins.

A deep comprehension of the regulation points of gene expression is crucial to understand the diverse cellular responses. Eukaryotic gene expression involves multiple exquisitely controlled regulatory layers such as chromatin accessibility, transcription and translation rates, RNA processing and RNA and protein stability. Although the main control point for several genes is at the transcriptional level, diverse post-transcriptional mechanisms also contribute to gene regulation and cell-specific expression patterns [63].

Growing evidence indicates that ROS function as crucial messengers in cell proliferation and survival by regulation of gene transcription [64]. Moreover, ROS can also modulate gene expression at post-transcriptional levels [65,66,67]. On the other hand, oxidative stress induces alterations in gene transcription, responding to ROS levels by the activation of a complex antioxidant defense system [64]. Redox-mediated mitochondria-nucleus crosstalk coordinates cellular metabolism with chromatin remodeling, gene expression, cell cycle, DNA repair and cell differentiation [68]. The expression of many components of the stress defense system is regulated through NRF2 [69]. Additionally, p53 plays a key role in mechanisms intended to repair ROS-induced damages [70]. Interestingly, during severe stress conditions, the induction of a very efficient ROS scavenging machinery by the NRF2-dependent response interferes with the pro-apoptotic response induced by p53. Regarding p53 pro-oxidant functions, time course experiments demonstrated the existence of a cascade in which p53 promotes the transcription of redox-controlling genes with the subsequent cellular ROS enhancement, that leads to mitochondrial oxidative damage and promotes apoptosis through a three-step process [71]. The balance between the pathways associated with these TFs determines the cellular fate, which is dependent on the redox status [31]. Particularly, p53 suppresses the NRF2-dependent transcription of genes whose regulator region contains antioxidant response elements, in several tumoral cell lines [72]. In turn, NRF2 negatively regulates p53 by promoting MDM2 transcription in murine embryonic fibroblasts [73].

Particularly in ES cells, redox homeostasis is essential for self-renewal and pluripotency maintenance, and also for proper differentiation. ROS accumulation may cause apoptosis, premature senescence and unscheduled differentiation [68]. The role of ROS in regulating stem cell dynamics has implications for various pathologies, including cancer and age-related diseases, further highlighting that fine regulation of ROS homeostasis plays an essential role in the fate decision of stem cells from adult tissues [18]. Notably, it was proposed that p53 regulates stem cells processes by acting as either an antioxidant or a pro-oxidant in a context-dependent manner. For example, under physiological conditions or mild oxidative stress, p53 is maintained at low levels by MDM2 mediated proteasomal degradation [74] and it induces the transcription of antioxidant genes, hence maintaining low ROS levels [75]. On the other hand, under persistent and severe oxidative stress conditions, p53 is hyper activated and exerts pro-oxidative activity. In this context, p53 promotes ES cells differentiation and certain pathways that safeguard DNA fidelity, thus preserving their genome integrity and avoiding the propagation of genetic aberrations to daughter cells [24,76]. Beyond ROS modulation by p53, this TF has a wide range of actions in stem cells. Genomic stability maintenance, induction of differentiation and regulation of metabolism are some examples of p53 functions [77]. Furthermore, unveiling p53 roles in pluripotent stem cells will contribute to the development of safe stem cells-based therapies.

Regarding the defense against oxidative damage, there is compelling evidence that propose HO-1 induction as an important cytoprotective mechanism and some authors explored HO-1 stability modulation. Hwang et al. showed that HO-1 forms dimers and oligomers in the ER and this interaction results in its stabilization [78]. The ubiquitination also plays a role in its stability, probably involved in its ER-associated proteasomal degradation [79]. A recent work demonstrates that 14-3-3ζ, a protein implicated in the initiation and progression of multiple types of cancer, interacts with HO-1 and regulates its stability by inhibiting its ubiquitination [80]. It is interesting to mention that there are not previous reports about regulators of HO-1 stability in ES cells. Our results showed that p53 KO ES cells have higher HO-1 protein levels compared to WT cells; however, similar levels of NRF2 and pS40 NRF2 were found in both cell lines, consistent with the outcome that no differences were detected in HO-1 mRNA levels. These findings along with the increased stability of HO-1 in KO cells revealed by CHX treatment, lead us to speculate that a negative post-transcriptional mechanism exerted by p53 is involved in HO-1 stability modulation, suggesting a new role for this protein in the antioxidant system of ES cells.

Work by us [56] and others [81] reported the localization of HO-1 in the nucleus of cancer cells, where it is suggested to mediate signaling functions. Even more, this localization may be associated with certain stress conditions like oxidative stress [57]. To our best knowledge, herein, for the first time, we report HO-1 nuclear localization in both WT and p53 KO ES cells lines. This is an interesting finding that encourages us to focus future work in exploring a putative HO-1 nuclear function in ES cells. Interestingly, p53 KO ES cells present slightly but significantly lower N/C ratio than WT ES cells, suggesting higher HO-1 cytoplasmic levels in the absence of p53. We hypothesize that this could be a compensatory mechanism due to the lack of p53, that could contribute to cellular homeostasis maintenance. As we mentioned before, HO-1 canonical activity takes place in the cytoplasm. Therefore, HO-1 higher levels in the cytoplasm of p53 KO ES cells could be responsible for the maintenance of redox balance. This idea is consistent with the fact that p53 KO ES cells have almost unaltered ROS levels and with the absence of HO-1 induction after H_2_O_2_ treatment in these cells. Finally, we detected that the regulation of the *Sod2* gene follows the same expression pattern as HO-1, since *Sod2* mRNA levels are similar between both cell lines but KO ES cells display higher SOD2 protein levels. This interesting observation suggests that p53 might also control the expression of other relevant antioxidant proteins at the post-transcriptional level. Future studies will be necessary to reveal a putative common p53-mediated mechanism involved in protein stability modulation, particularly of those participating in the antioxidant defense system.

Regarding p53 and HO-1 gene regulation, Nam et al. showed that specifically under oxidative stress, but not under DNA damage-associated stress, p53 promotes cell survival by induction of *Hmox1* transcription [29]. In line with this report, we found that HO-1 gene is induced by H_2_O_2_ treatment only in WT ES cells. Remarkably, no *Hmox1* induction was observed in p53 KO ES cells, due to the lack of p53 and in accordance with Nam et al. findings in mouse embryonic fibroblasts and in human colon carcinoma cells [29]. However, we cannot rule out that these cells are insensitive to H_2_O_2_ treatment due to their high endogenous HO-1 levels. We propose that these basal levels would be a consequence of compensatory cellular mechanisms triggered by the high oxidative stress, which boosted HO-1 levels counteracting altered redox homeostasis.

In addition, during ES cells differentiation both HO-1 [37,58,60,82,83,84] and p53 [24,76,82,85] play a relevant role. Using the Chip Atlas platform, we found that in undifferentiated ES cells *Hmox1* is associated with negative transcriptional regulators. Further, we found histone modifications known to be associated with gene repression in undifferentiated ES cells. Moreover, in differentiation-inducing conditions, histone marks related to gene activation were revealed. These observations are consistent with our results that show the increment of HO-1 during the differentiation process. Furthermore, analysis of microarray and RNA-seq experiments from publicly available data bases allowed us to analyze HO-1 expression during both direct and non-direct differentiation protocols. Using data from non-directed and lineage-specific differentiation protocols we revealed that HO-1 is modulated during diverse differentiation processes. Interestingly, despite its higher basal levels, HO-1 also increases during differentiation in p53 KO ES cells. The fact that HO-1 gene is induced during differentiation, contrary to some other antioxidant proteins like SOD2, which expression decreases during this process, highlights the notion that besides being involved in cell stress defense, HO-1 could be a relevant protein for ES cells differentiation.

To conclude, in this work we report a regulatory relationship between two main components of different cellular defense systems in ES cells, opening new perspectives to delve into the complex mechanisms involved in ROS balance that are crucial for the maintenance of stem cells’ fundamental properties and for their differentiation.

## Figures and Tables

**Table 1:** Expression microarray and RNA-seq studies selected from the Stemformatics platform (https://www.stemformatics.org./). N/A = not available.

	Reference	Cell Line	Platform	Accession No
Non-directed diff	Enver et al., 2005 [42]	H7	Affymetrix U133A	N/A
Reprograming	Kyrkou et al., 2016 [43]	Fibroblast and iPS cells	Affymetrix HuGene-1_0-ST V1	GSE58932
Embryo development	Yi et al., 2010 [44]	Human embryos	Affymetrix HG-U133_Plus_2	GSE15744
Ectodermal diff	Pandya et al., 2017 [45]	iPS cells and iPS derived microglia	Affymetrix MoGene-1_0-ST V1	GSE47605
Ectodermal diff	Mariani et al., 2015 [46]	iPS cells	Illumina HiSeq 2000	GSE61476
Mesodermal diff	An et al., 2014 [47]	Erythroblasts	Illumina HiSeq 2000	GSE53983
Mesodermal diff	Granchi et al., 2010 [48]	Mesenchymal stem cells	Affymetrix HG-U133_Plus_2	GSE12267
Endodermal diff	Si-Tayeb et al., 2010 [49]	iPS cells	Affymetrix HG-U133_Plus_2	GSE14897
Endodermal diff	Teo et al., 2011 [50]	ES cells	Illumina HumanRef-8 V3	E-MTAB-467

## Data Availability

Data sharing not applicable.

## Supplementary Materials

The following are available online at https://www.mdpi.com/2073-4409/10/1/35/s1, Figure S1: Restoring p53 expression in p53 KO ES cells, Figure S2: Hemin induction of HO-1 expression and cycloheximide effect, Figure S3: HO-1 immunofluorescence visualized by confocal microscopy. Figure S4: Analysis of public data from ChIP Atlas and Stemformatics platforms.
