# Supplementary material for: Novel Interplay between p53 and HO-1 in Embryonic Stem Cells
Source: Cells. 2020 Dec 29;10(1):35. doi: 10.3390/cells10010035 (PMC7823265; doi:10.3390/cells10010035)
Supplement: Supplementary file 1 [file cells-10-00035-s001.pdf]

Supplementary Material

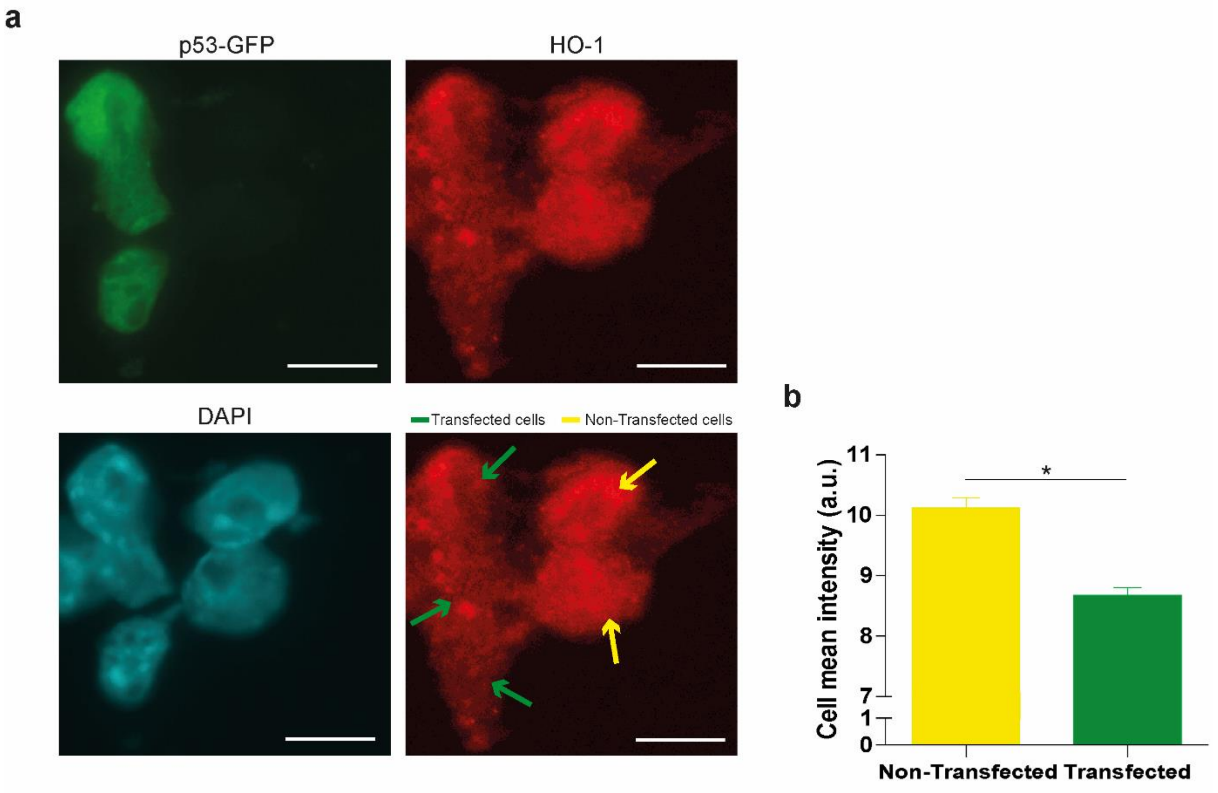

Supplementary Figure S1. Restoring p53 expression in p53 KO ES cells. p53 KO ES cells were transfected with an expression vector encoding a p53-GFP fusion protein. 48 h after transfection, HO-1 was visualized by immunofluorescence and nuclei were counterstained with DAPI. Transfected cells were identified by GFP fluorescence detection. (**a**) Representative images of transfected and non-transfected cells. In the right lower panel thin green arrows indicate transfected cells and thick yellow arrows indicate non-transfected cells. Scale bars: 10 μm. (**b**) Representative images of HO-1 immunofluorescence intensities depicted in (a) were quantified, bars represent the mean ± SEM (*, $p < 0.05$). HO-1 intensity signal is lower in transfected cells compared to non-transfected cells, evidencing HO-1 downregulation when p53 expression is restored.

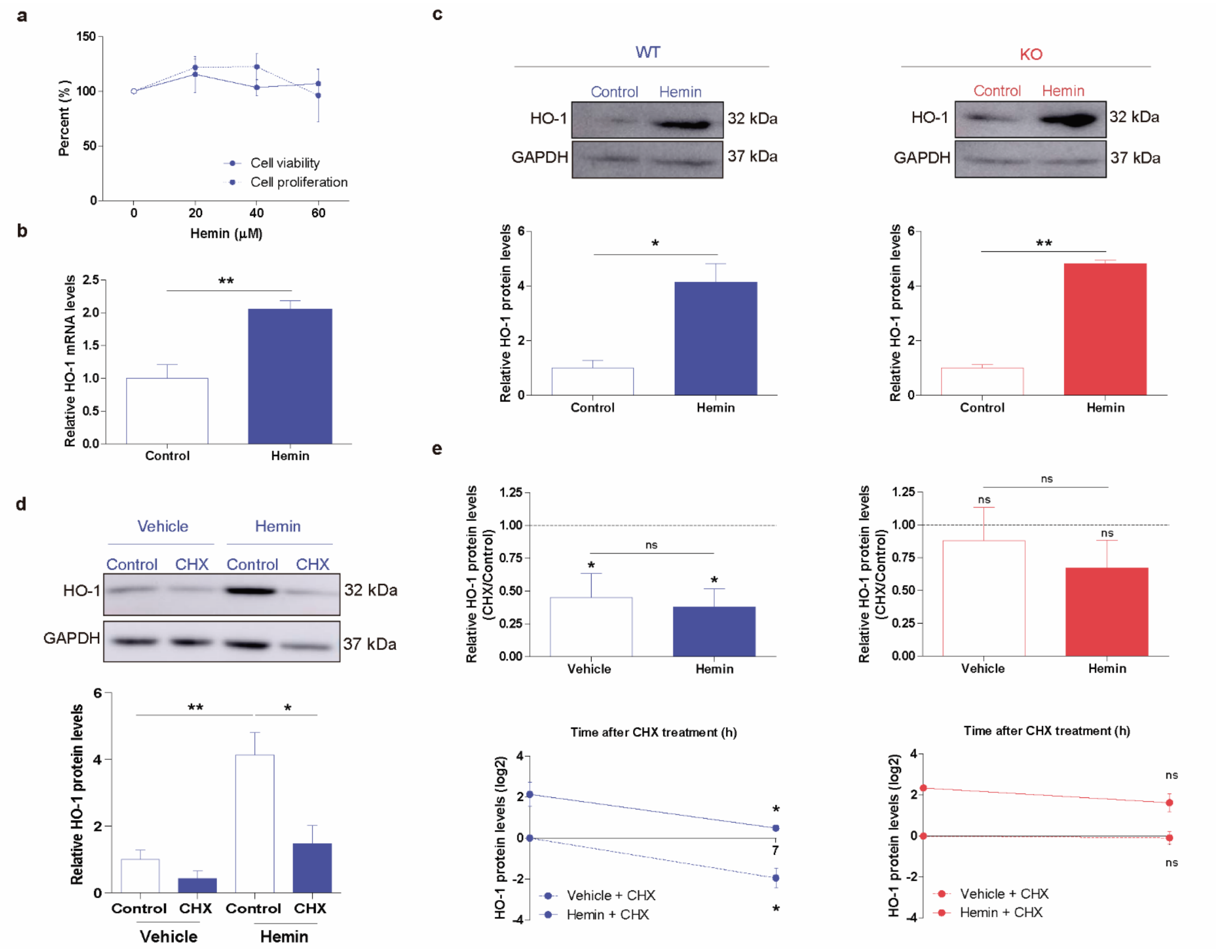

Supplementary Figure S2. Hemin induction of HO-1 expression and cycloheximide effect. (**a**) WT ES cells were cultured in the presence of 0, 20, 40, 60 µM hemin during 20 h and cell viability and proliferation were assayed. (**b**) WT ES cells were treated or not with 20 µM hemin during 20 h. HO-1 mRNA levels were analyzed by RT-qPCR, normalized to the housekeeping gene *Gapdh*. (**c**) WT and p53 KO ES cells were treated or not with 20 µM hemin during 20 h and HO-1 protein levels were analyzed by Western blot. Upper panels show representative blots. GAPDH was used as loading control. Lower panels show HO-1/GAPDH band densitometry. (**d**) WT ES cells were pre-treated with 20 µM hemin or DMSO (vehicle) during 20 h and after treated without (control) or with 10 µM cycloheximide (CHX) during 7 h. HO-1 levels were analyzed by Western blot. Upper panels show representative blots. GAPDH was used as loading control. Lower panels show HO-1/GAPDH band densitometry. (**e**) The ratio of HO-1 protein levels in cycloheximide treated ES cells (CHX) respect to the control conditions without CHX (vehicle or hemin) is shown in upper panels. Asterisks indicate significant differences between cells treated with CHX respect to the control conditions without CHX (vehicle or hemin). Non-significant (ns) differences were found in HO-1 protein levels of WT or KO ES cells pre-treated with hemin respect to vehicle pre-treated ES cells. In lower panels, time-dependent HO-1 expression plots are shown. Bars represent the mean ± SEM of three independent experiments. Significant differences: *, $p < 0.05$; **, $p < 0.01$.

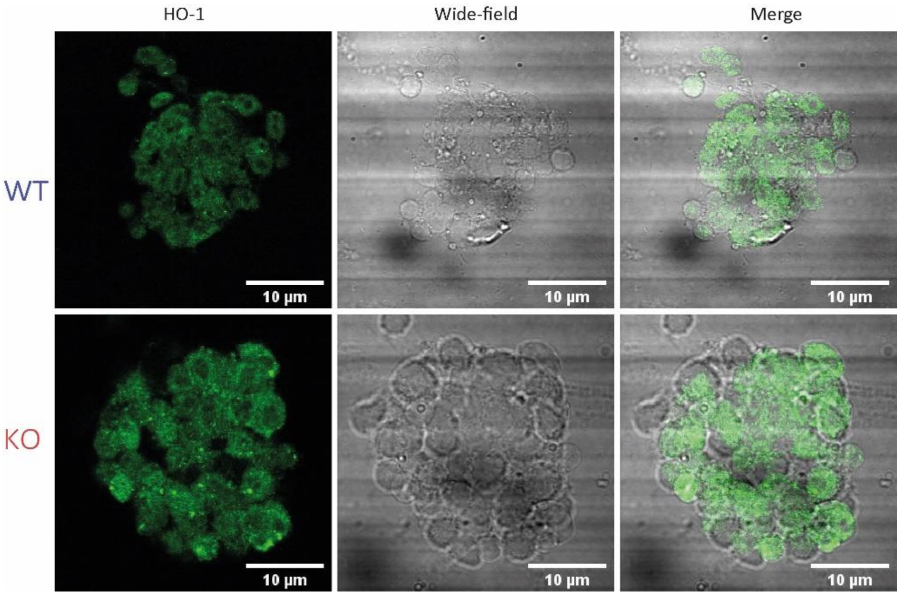

Supplementary Figure S3. HO-1 immunofluorescence visualized by confocal microscopy. HO-1 levels were analyzed by immunofluorescence in WT and KO ES cells. Representative confocal images of ES cell colonies and the merged with the corresponding transmission images are shown. The green pseudocolor represents HO-1 specific immunostaining, Scale bars: 10 µm.

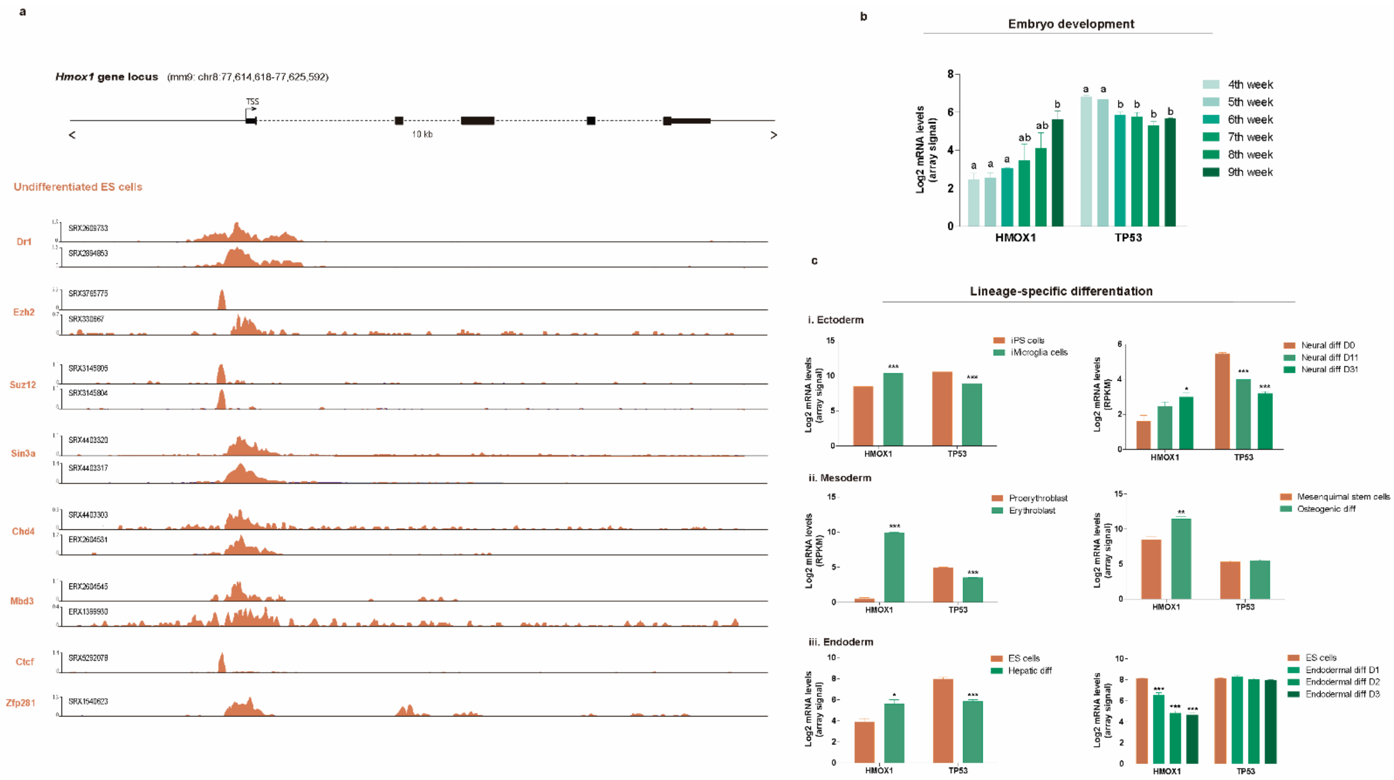

Supplementary Figure S4. Analysis of public data from ChIP Atlas and Stemformatics platforms. (**a**) Analysis of the binding of multiple transcriptional regulators to *Hmox1* locus in undifferentiated ES cells and ES cells subjected to differentiation protocols (differentiated cells) using Chip Atlas data-mining platform. Dataset identification numbers of each experiment are shown at the top of the y-axis. (**b**) Microarray data analysis of HO-1 and p53 mRNA levels during human embryo development (GSE15744). Bars represent the mean ± SEM of each experiment. Different letters indicate significant differences between conditions corresponding to the indicated week of embryo development. (**c**) Analysis of *HMOX1* and *TP53* gene expression from the indicated human cell lines subjected to directed differentiation to specific lineages: i, ectoderm (GSE47605 and GSE61476); ii, mesoderm (GSE53983 and GSE12267); iii, endoderm (GSE14897 and E-MTAB-467). Bars represent the mean ± SEM of each experiment. Asterisks indicate significant differences between undifferentiated (orange) and differentiated (green) cells (*, $p < 0.05$; **, $p < 0.01$; ***, $p < 0.001$).
